# The Relevance of Curative‐Intent Metastasectomy in Colorectal Cancer Patients: Real‐World Insights From a Certified Comprehensive Cancer Center in Germany

**DOI:** 10.1002/ijc.70540

**Published:** 2026-05-09

**Authors:** Julian W. Holch, Jo‐Ann Eisenburger, Antony Weber, Iris Kammermeier‐Wehlau, Yannick Wiberg, Fabian Allmendinger, Kathrin Heinrich, Lena Weiss, C. Benedikt Westphalen, Victoria Krenmayr, Klara Dorman, Danmei Zhang, Theres Fey, Karin Berger‐Thürmel, Ulrich Wirth, Florian Kühn, Michael von Bergwelt‐Baildon, Jens Werner, Volker Heinemann

**Affiliations:** ^1^ Department of Medicine III University Hospital, LMU Munich Munich Germany; ^2^ Comprehensive Cancer Center (CCC Munich^LMU^) LMU University Hospital Munich Munich Germany; ^3^ German Cancer Consortium (DKTK) Partner Site Munich and German Cancer Research Center (DKFZ) Heidelberg Germany; ^4^ Bavarian Cancer Research Center (BZKF) Munich Germany; ^5^ Department of Radiation Oncology Heidelberg University Hospital, Heidelberg University Heidelberg Germany; ^6^ National Center for Radiation Research in Oncology (NCRO) Heidelberg Institute for Radiation Oncology (HIRO) Heidelberg Germany; ^7^ National Center for Tumor Diseases (NCT) NCT Heidelberg, a Partnership Between DKFZ and Heidelberg University Hospital Heidelberg Germany; ^8^ Department of Radiation Oncology, Heidelberg Ion‐Beam Therapy Center (HIT) Heidelberg University Hospital, Heidelberg University Heidelberg Germany; ^9^ Department of General, Visceral and Transplantation Surgery University Hospital, LMU Munich Munich Germany

**Keywords:** metastasectomy, metastatic colorectal cancer, prognosis, real‐word evidence, resection

## Abstract

Long‐term survival in metastatic colorectal cancer (mCRC) can be achieved by curative‐intent metastasectomy. Patients ineligible for upfront (primary) metastasectomy (PM) may become candidates for secondary metastasectomy (SM) following response to primary chemotherapy (PCT), still with curative intent. Most studies focus either on resectable or unresectable mCRC, limiting insights into differential subgroup benefits. Here, real‐world data offer a comprehensive evaluation of PM, SM, and PCT. We analyzed real‐world data from mCRC patients treated at the certified Comprehensive Cancer Center of the LMU Hospital (CCC Munich^LMU^) from 2007 to 2021, following ESMO's guidance for real‐world evidence (GROW). Overall survival (OS) data were matched with the Bavarian Cancer Registry. OS was assessed using Kaplan–Meier estimates and Cox regression analysis, adjusting for prognostic factors, including primary tumor sidedness and number of metastatic sites. Of 840 evaluable patients, 166 (20%) underwent PM, 520 (62%) received PCT, and 109 (13%) became eligible for SM after response to systemic treatment. OS was significantly longer for PM versus PCT (82.3 vs. 41.6 months, HR = 0.47, *p* < 0.0001). Multivariate analysis confirmed PM benefit across all subgroups, including high‐risk patients (e.g., right‐sided and multisite mCRC). OS was comparable between PM and SM (82.3 vs. 80.9 months), whereas patients ineligible for local treatments had the worst OS (25 months). Both PM and SM were associated with excellent OS, also for patients with high‐risk mCRC. Our findings underscore the importance of initial evaluation and continuous reassessment of resectability throughout the course of treatment against mCRC, as implemented at the CCC Munich^LMU^.

## What's New?

Patients with metastatic colorectal cancer who are ineligible for upfront metastasectomy may still become candidates for secondary metastasectomy with curative intent following response to primary chemotherapy. However, most studies dichotomize metastatic colorectal cancer into resectable and unresectable disease. This large real‐world analysis shows that both primary and secondary curative‐intent metastasectomy in metastatic colorectal cancer achieve excellent and comparable long‐term survival. By linking institutional data with registry‐validated outcomes, the study demonstrates survival benefits even in high‐risk subgroups. The findings highlight the importance of repeated multidisciplinary reassessment of resectability and support broad adoption of multidisciplinary team‐guided surgical strategies in routine oncology practice.

## Graphical abstract

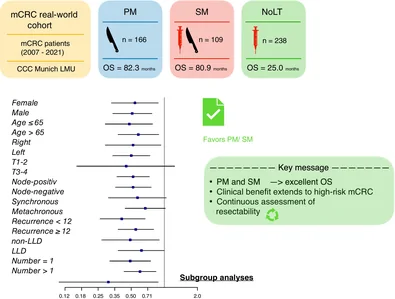

**Abbreviations**
5y: 5 years
BRAF: v‐raf murine sarcoma viral oncogene homolog B1
CI: confidence interval
CRC: colorectal cancer
EGFR: epidermal growth factor receptor
GROW: ESMO's Guidance for Real‐World Evidence
HR: hazard ratio
KRAS: Kirsten rat sarcoma viral oncogene homolog
LLD: liver‐limited disease
mCRC: metastatic colorectal cancer
MDT: multidisciplinary team
n.r.: not reported
NoLT: no local treatment
nPM: primary metastectomy without (documented) curative intent
NRAS: neuroblastoma RAS viral oncogene homolog
nSM: secondary metastectomy without (documented) curative intent
OS: overall survival
PCT: primary chemotherapy
PIT: primary interventional treatment
PM: primary metastectomy in curative intent
PS: post‐resection survival
pts.: patients
RAS: rat sarcoma
SIT: secondary interventional treatment
SM: secondary metastectomy in curative intent
STROBE: Strengthening the Reporting of Observational Studies in Epidemiology guidelines
VEGF: vascular endothelial growth factor

## 1 Introduction

Colorectal cancer (CRC) is the third most commonly diagnosed cancer worldwide, with an estimated 1.9 million new cases in 2022, and ranks as the second leading cause of cancer‐related mortality, accounting for approximately 904,000 deaths [1]. Around 25% of patients present with synchronous metastasis at the time of diagnosis, while an additional 18%–25% develop metachronous metastasis within five years [2, 3, 4, 5]. The most common metastatic sites are the liver, followed by lungs and peritoneum [3, 5]. Predominantly single‐site mCRC, most frequently affecting the liver (40%) and lungs (15%), is amenable to radical metastasectomy with curative intent, achieving 5‐year survival rates of up to 60% [3, 5, 6, 7, 8]. However, up to 80% of patients initially present with multisite metastases or anatomically irresectable disease and are managed with systemic therapy [3]. Despite advancements in chemotherapy, 5‐year overall survival (OS) rates remain below 20% for non‐resected patients, whereas resection rates in specialized centres vary widely between 7% and 84% [1, 3].

For initially unresectable patients, secondary resection can be achieved after response to upfront (conversion) chemotherapy and is feasible in up to 58% of patients, still with curative intent [3, 9]. Here, the role of structured resectability assessment and repeated multidisciplinary team (MDT) evaluation has gained increasing importance, as studies suggest that non‐specialist oncologists tend to underestimate resectability compared to expert surgical teams [3, 10]. Consequently, the German Cancer Society mandates the implementation of MDTs in certified Comprehensive Cancer Centers (CCCs) to ensure interdisciplinary treatment of patients with gastrointestinal tumors.

While extensive research has focused on liver‐limited disease (LLD) and lung‐limited disease, data on multisite resections remain scarce [3]. Furthermore, most studies dichotomize mCRC into resectable and unresectable disease, without accounting for secondary resections or changes in resectability over time [10, 11, 12, 13]. Here, real‐world data provide a unique opportunity to evaluate the role of curative‐intent metastasectomy across different metastatic sites and patient subgroups and enable direct comparisons between patients undergoing primary metastasectomy (PM), primary chemotherapy (PCT), and secondary metastasectomy (SM).

This study leverages real‐world data from a CCC certified by the German Cancer Society to comprehensively evaluate curative‐intent metastasectomy across all metastatic sites and to compare survival outcomes among patients undergoing PM, PCT, and SM. A particular focus is placed on subgroup analysis considering a less established benefit of metastasectomy in high‐risk patients (e.g., with right‐sided primary tumor or more than one metastatic site).

Using a previously characterized and expanded cohort of uniformly treated mCRC patients at the Comprehensive Cancer Center of the LMU Hospital (CCC Munich^LMU^) [5], we further integrated matched survival data from the governmental Bavarian Cancer Registry and adhered strictly to ESMO's Guidance for Reporting Oncology Real‐World Evidence (GROW) [14], ensuring a robust and well‐documented data foundation for outcome evaluation.

## 2 Methods

### 2.1 Study Design

This explanatory, single‐center cohort study analyzed real‐world data from patients treated for proven metastatic colorectal adenocarcinoma at CCC Munich^LMU^, Germany. The study was designed according to the Strengthening the Reporting of Observational Studies in Epidemiology guidelines (STROBE) for retrospective cohort studies [15].

The routinely collected electronic health record data used in this research project were obtained in compliance with applicable data protection regulations and institutional governance policies. The data are owned and managed by LMU University Hospital, which granted access for research purposes. Data governance was ensured through established protocols for secure handling, and all data were anonymized before analysis to protect patient confidentiality.

### 2.2 Data Collection

Patients whose metastases were diagnosed between 01.01.1998 and 30.06.2021 with the following ICD‐10 codes were identified in the clinical information system (i.s.h.med) of the LMU University Hospital and included in the documentation: malignant neoplasm of the colon (C18.0–C18.9), malignant neoplasm of the rectosigmoid junction (C19) and malignant neoplasm of the rectum (C20). Patients with appendiceal neoplasms were excluded. Patients without any metastatic disease were documented but not included in further statistical analysis. The documentation and reporting of routinely collected electronic health record data within i.s.h.med followed the ESMO Guidance for Reporting Oncology Real‐World Evidence (GROW) [14]. Corresponding health data were collected and anonymized between September 15, 2021 and April 30, 2022. Information on demographics, clinicopathological parameters and treatment was collected: sex (male, female), age (in years), primary tumor location (left vs. right), T‐stage (T1–4), N‐stage (N0–2), molecular biomarkers (presence of activating mutation or wild‐type status of oncogenes *KRAS*, *NRAS*, *BRAF* and microsatellite instability status), stage of disease at first presentation (UICC I–IV), development of metastasis (synchronous or metachronous, the cut‐off between those two groups was 92 days after diagnosis of the primary tumor), location of metastasis (liver, lungs, peritoneal, others) at first diagnosis of metastatic disease, time to recurrence (in months), treatment of the primary tumor and of all diagnosed metastases (resection vs. type of local treatment). Data were assessed from laboratory, radiological, pathological and medical records identified in i.s.h.med.

### 2.3 Quality Assurance

To mitigate potential biases inherent to routinely collected health data analyses, particularly selection bias, information bias, and missing data bias, all patients treated at LMU hospital for the intended indication were systematically included. Furthermore, data collection was conducted at LMU University Hospital Medical Department III, which is certified under DIN ISO 9001 ensuring adherence to a structured quality management system to enhance data accuracy and reliability. Additionally, independent quality control of the data set was performed (by A.W., Y.W., and I.K.‐W.) to assess completeness, homogeneity, and duplicate records resulting in an error rate of below 5% based on random sampling of 10% of all patients [16].

To reduce the number of censored survival times, patient mortality data were cross‐referenced with records from the Bavarian State Cancer Registry, which permits data access for treating institutions under established legal and regulatory frameworks. In cases of incongruence, registry‐derived OS data superseded clinically available data in our cohort. The latest data acquisition was conducted at June 30, 2022.

Only patients with curative intent resection have been counted for PM and SM. Consequently, patients without clearly documented curative intention as well as those treated with locoregional or local‐ablative modalities were excluded due to the inability to reliably assess completeness of local therapy, especially within a retrospective study design.

### 2.4 Statistical Analysis

The primary end point of this study was OS, which was calculated from the date of diagnosis of first metastasis until the date of last follow‐up or death. The date of diagnosis was based on the last diagnostic procedure initiating management for mCRC. To avoid lead time bias when comparing primary and secondary resection, survival after metastasectomy (post‐resection survival; PS) was further calculated from the date of metastasectomy until the date of last follow‐up or death. Fisher's exact test and chi square test were applied to compare dichotomous and continuous variables. To analyze survival, the Kaplan–Meier method and Cox regression analysis were implemented. OS was compared through log‐rank tests. The following parameters were considered as potentially prognostic factors and integrated into multivariate Cox regression analysis: gender, age, primary tumor location, T‐stage of primary tumor, N‐stage of primary tumor, development of metastasis (synchronous vs. metachronous), disease‐free interval, LLD status, and number of metastatic sites. Pathological T‐ and N‐staging was used when available; otherwise, clinical staging was applied with a uniform staging prefix for both T and N categories. The results of multivariate Cox regressions are displayed via forest plots. All tests were performed two‐sided, and the *p* value indicating statistical significance was considered at 0.05. Statistical analysis was conducted with Jamovi and RStudio (Version 2023.12.1+402).

## 3 Results

### 3.1 Patient Population

**FIGURE 1:**
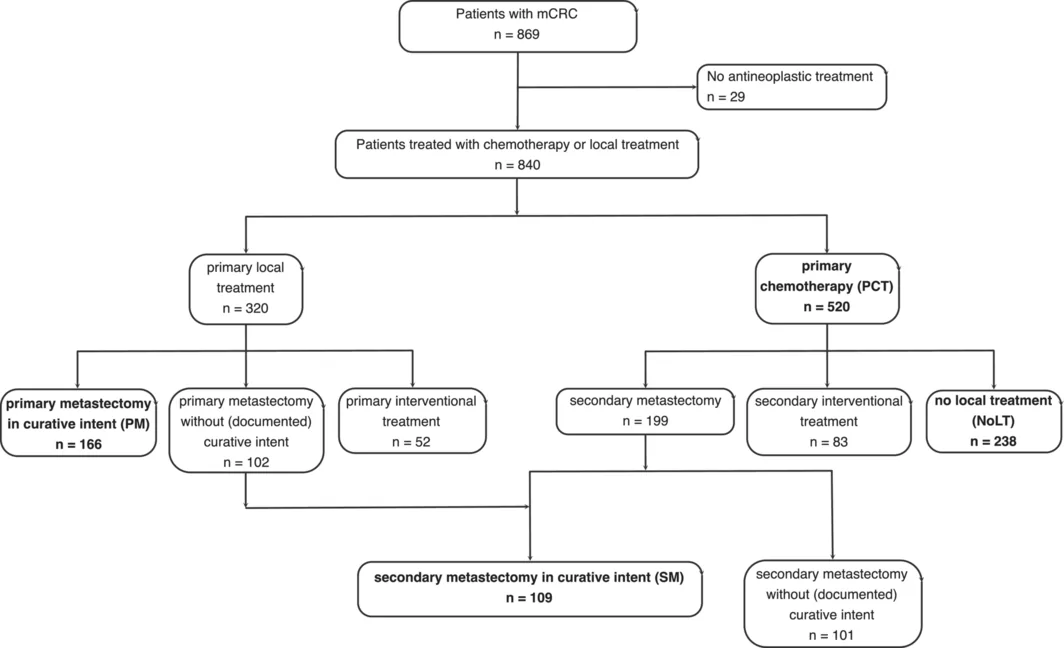
CONSORT diagram. mCRC, metastatic colorectal cancer; NoLT, no local treatment; PCT, primary chemotherapy; PM, primary metastectomy in curative intent; SM, secondary metastectomy in curative intent.

Of 869 identified patients with mCRC, 29 (3%) were excluded as they did not receive any treatment for metastatic disease (Figure 1). Within the patient cohort of 840 patients, 520 patients (62%) were initially identified as not amenable to metastasectomy or local treatment upon case discussion in MDT and started upfront chemotherapy (PCT). In 320 patients, upfront local treatment was performed with 166 patients (20%) receiving curative‐intent metastasectomy (PM). Fifty two patients (6%) were initially treated with local‐ablative or locoregional treatment. One hundred two patients (12%) underwent upfront surgery without clearly documented curative intent. As subgroup assignment strictly relied on a priori treatment intention, patients without available information on curative intent before surgery were excluded. In most cases, primary resection was performed externally without sufficient documentation. In 11 patients (1%), two‐staged metastasectomy was performed with curative intent. In patients initially treated with chemotherapy, 199 patients (24%) underwent surgery of whom 98 patients (12%) were secondarily resected with curative intent (SM). Including patients after repeated resection, a total of 109 patients received SM. In patients operated after PCT, resection without curative intent was performed with residual metastasis in 70 patients (8%). In 29 patients (3%), curative intention could not be confirmed due to external treatment and insufficient documentation. In patients treated upfront with chemotherapy, 238 patients (28%) were not amenable to local treatment options throughout the course of disease (NoLT) and 83 patients (16%) were treated with local interventions (Figure 1).

### 3.2 Patient Characteristics

Baseline demographic and clinicopathological characteristics of the evaluated subgroups are presented in Table 1. Patients treated with PM tended to be older than those treated with PCT. Right‐sided primary tumors were more prevalent in patients receiving PCT. *RAS* mutation analysis was performed in 66.3% and 86% of patients treated with PM and PCT, respectively, whereas *BRAF* mutation analysis was performed in 48,8% and 63.3% of patients treated with PM and PCT, respectively. Activating mutations in *BRAF* and *RAS* oncogenes were well balanced among subgroups. Regarding tumor stage, patients treated with PM were more frequently diagnosed with lower T‐stage, N‐stage and UICC stage. Furthermore, metachronous metastasis with a disease‐free interval of ≥ 12 months and lower number of metastatic sites were enriched in patients with PM, whereas LLD status was well balanced in both subgroups.

**TABLE 1:** Baseline characteristics.

	Total cohort (*n* = 840), *N* (%)	PM (*n* = 166), *N* (%)	PCT (*n* = 520), *N* (%)	*p*
*Gender*				
Male	503 (59.9)	101 (60.8)	322 (61.9)	0.803
Female	337 (40.1)	65 (39.2)	198 (38.1)	
*Age*				
≤ 65	418 (49.8)	77 (46.4)	283 (54.4)	0.071
> 65	422 (50.2)	89 (53.6)	237 (45.6)	
*Primary tumor location*				
Right sided	221 (26.3)	31 (18.7)	136 (26.2)	**0.036**
Left sided	592 (70.5)	129 (77.7)	369 (71.0)	
Right and left sided	17 (2.0)	2 (1.2)	12 (2.3)	
n.r.	10 (1.2)	4 (2.4)	3 (0.610)	
*RAS*				
Wildtype	366 (43.6)	62 (37.3)	241 (46.3)	**< 0.001**
Mutation	310 (36.9)	48 (28.9)	206 (39.6)	
n.r.	164 (19.5)	56 (33.7)	73 (14.0)	
*BRAF*				
Wildtype	461 (54.9)	72 (43.4)	301 (57.9)	**0.003**
Mutation	45 (5.4)	9 (5.4)	28 (5.4)	
n.r.	334 (39.8)	85 (51.2)	191 (36.7)	
*Initial stage (UICC)*				
I	32 (3.8)	13 (7.8)	13 (2.5)	**< 0.001**
II	86 (10.2)	26 (15.7)	35 (6.7)	
III	158 (18.8)	48 (28.9)	78 (15.0)	
IV	537 (63.9)	68 (41.0)	383 (73.7)	
n.r.	27 (3.2)	11 (6.6)	11 (2.1)	
*T‐stage of primary*				
T1–T2	78 (9.3)	24 (14.4)	43 (8.2)	**< 0.001**
T3–T4	640 (76.2)	132 (79.5)	381 (73.3)	
Tx	12 (1.4)		11 (2.1)	
n.r.	110 (13.1)	10 (6.0)	85 (16.3)	
*Primary tumor*				
Node‐negative	208 (24.8)	51 (30.7)	113 (21.7)	**< 0.001**
Node‐positive	485 (57.7)	100 (60.2)	294 (56.5)	
n.r.	147 (17.5)	15 (9.0)	113 (21.7)	
*Development of metastasis*				
Metachronous	303 (36.1)	98 (59.0)	137 (26.3)	**< 0.001**
Synchronous	537 (63.9)	68 (41.0)	383 (73.7)	
*Disease‐free‐interval*				
< 12	564 (64.9)	103 (62.0)	339 (65.2)	**< 0.001**
≥ 12	189 (21.7)	63 (38.0)	81 (15.6)	
n.r.	116 (13.3)		100 (19.2)	
*Number of the affected organs*				
1	592 (68.1)	152 (91.6)	322 (61.9)	**< 0.001**
> 1	277 (31.9)	14 (8.4)	198 (38.1)	
*LLD status*				
Non‐LLD	462 (53.2)	81 (48.8)	275 (52.9)	0.359
LLD	407 (46.8)	85 (51.2)	245 (47.1)	

*Note:* Statistically significant results are displayed in bold.

Abbreviations: BRAF, v‐raf murine sarcoma viral oncogene homolog B1; LLD, liver‐limited disease; n.r., not reported; PCT, primary chemotherapy; PM, primary metastectomy in curative intent; RAS, rat sarcoma.

### 3.3 Metastatic Patterns

Liver metastases were observed in the majority of patients (71.9%), with liver‐limited disease being most common among those who underwent upfront surgery (51.2% vs. 47.1%). The second most frequent site of metastases was the lungs, with lung‐limited disease occurring more often in the PM subgroup compared to PCT (11.4% vs. 4.4%). Less common metastatic sites included the ovaries (3.2%), bones (3.1%), and brain (1.5%).

### 3.4 Treatment Patterns

PCT consisted of standard doublet or triplet combination regimens in 454 patients (87.3%) compared to 50 patients (9.6%) who received mono chemotherapy and 16 receiving other regimens, for example, experimental treatment (Table S2). Three hundred eighty‐seven patients (74.4%) received chemotherapy in conjunction with a monoclonal antibody either targeting vascular endothelial growth factor (VEGF; 47.5%) or epidermal growth factor receptor (EGFR; 26.9%).

PM consisted of liver resection in 93 patients (56.0%), peritonectomy in 32 patients (19.3%), and lung resection in 20 patients (10.2%) of patients (Table S3). R0/1‐resection status could be verified in 129 patients, with R0‐resection documented in 119 patients (92.2%) and R1‐resections in 10 patients (7.8%).

SM consisted of liver resection in 89 patients (81.7%), lung resection in 7 patients (6.4%), and peritonectomy in 4 patients (3.7%) (Table S4). Combined resections were performed in 7 patients (6.4%). R0/1‐resection status could be verified in 93 patients with R0‐resection documented in 87 patients (93.5%) and R1‐resections in 6 patients (6.5%).

### 3.5 OS According to Primary Treatment

**FIGURE 2:**
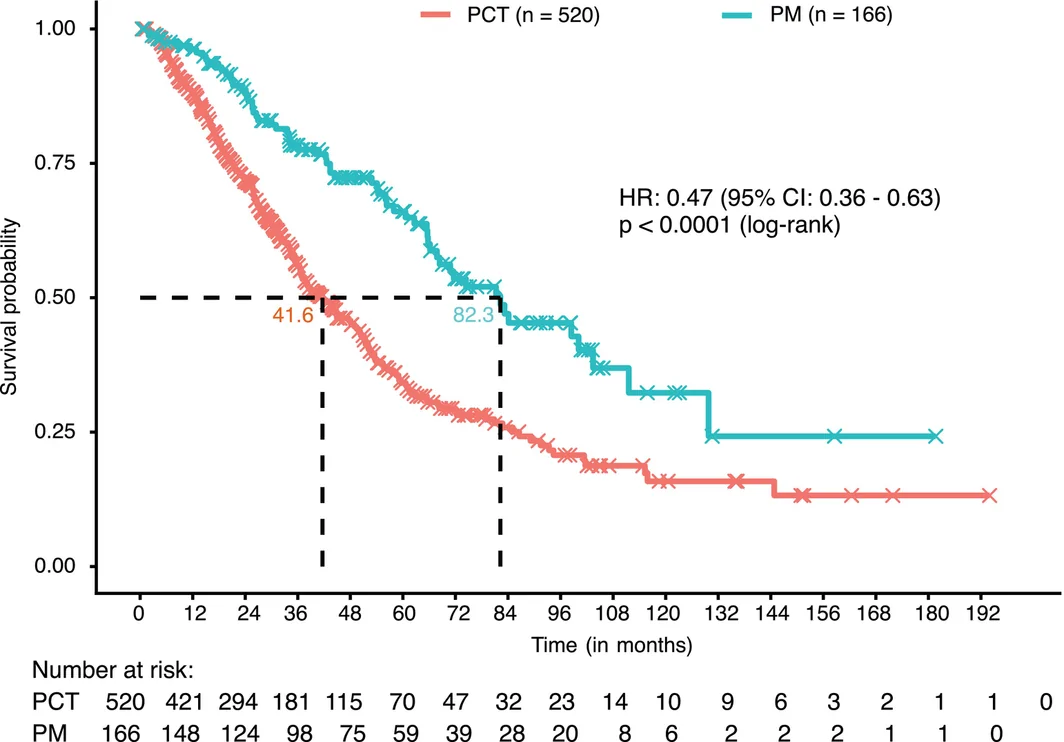
Overall survival by initial treatment. CI, confidence interval; HR, hazard ratio; PCT, primary chemotherapy; PM, primary metastectomy in curative intent.

After a median follow‐up of 63.7 months for patients with PM and 45.0 months for patients with PCT, OS was significantly longer in patients who underwent PM compared to patients treated with PCT: 82.3 versus 41.6 months; HR = 0.47, 95% CI: 0.36–0.63; *p* < 0.0001 (Figure 2). The 5‐year survival rate was 66.1% for patients undergoing PM, compared to 34.2% for those receiving PCT.

### 3.6 Subgroup Benefits of PM vs PCT

**FIGURE 3:**
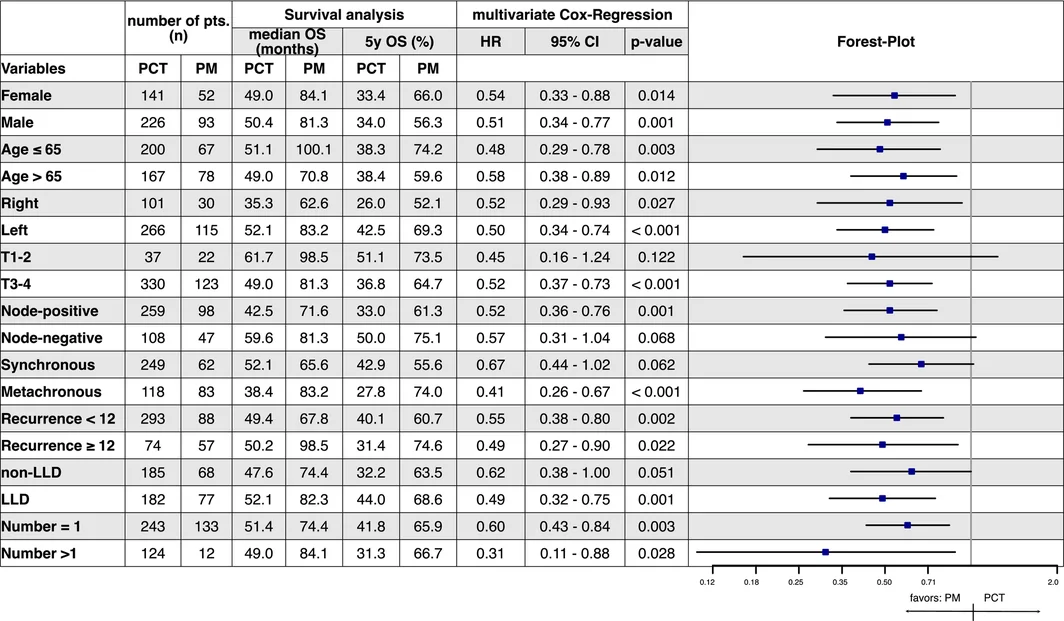
Forest plot of subgroup analyses comparing overall survival between treatment modalities (PCT vs. PM). HRs with 95% CIs were derived from multivariable Cox proportional hazards regression models performed separately within each predefined subgroup. All Cox regression models were adjusted for the full set of covariates within the respective subgroup. Median overall survival (OS, months) and 5‐year OS rates (%) for each treatment group are provided for descriptive purposes. An HR < 1 indicates a survival benefit in favor of PM. 5y, 5 years; CI, confidence interval; HR, hazard ratio; LLD, liver‐limited disease; OS, overall survival; PCT, primary chemotherapy; PM, primary metastectomy in curative intent; pts., patients.

In multivariate Cox regression analysis, all evaluated patient subgroups exhibited improved OS when eligible for PM compared to PCT (Figure 3). This survival benefit was also evident in patients with high‐risk features, specifically right‐sided primary tumors (HR = 0.52, 95% CI: 0.29–0.93, *p* = 0.027), synchronous metastases (HR = 0.67, 95% CI: 0.44–1.02, *p* = 0.062), disease‐free interval < 12 months (HR = 0.55, 95% CI: 0.38–0.80, *p* = 0.002), and patients with more than one metastatic site (HR = 0.31, 95% CI: 0.11–0.88, *p* = 0.028). Of note, the survival benefit observed in patients with synchronous metastases did not reach statistical significance (*p* > 0.05).

### 3.7 Comparison of PM vs. SM

**FIGURE 4:**
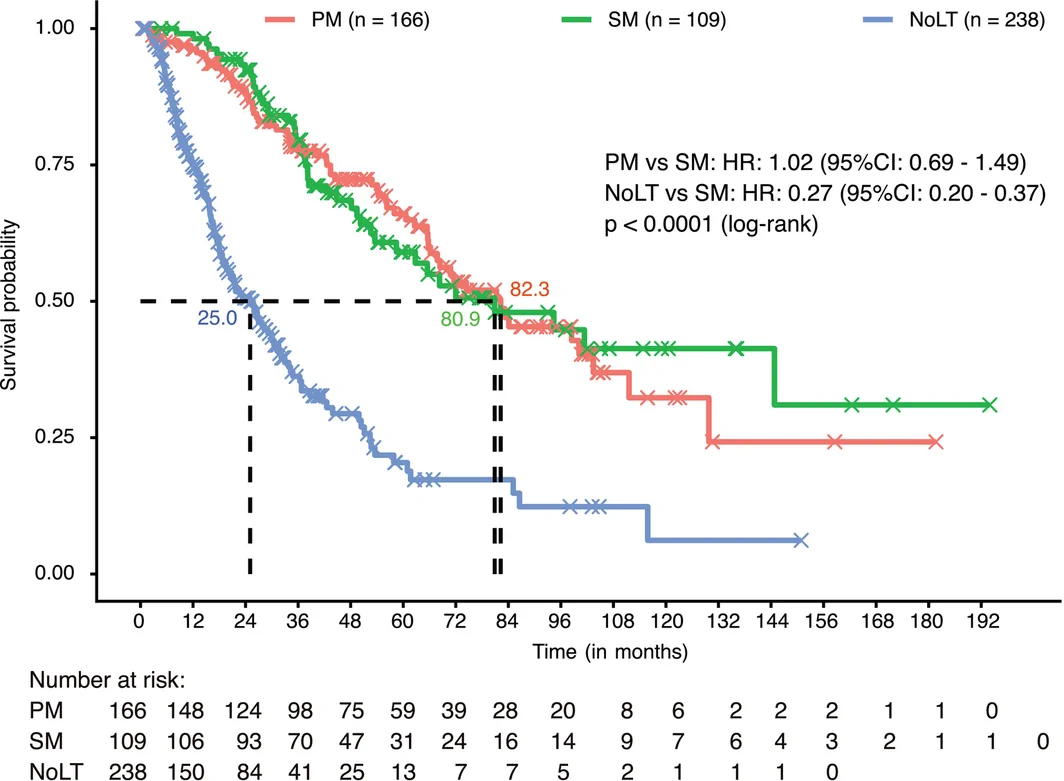
Overall survival by eligibility for metastectomy in curative intent. CI, Confidence interval; HR, hazard ratio; NoLT, no local treatment; PM, primary metastectomy in curative intent; SM, secondary metastectomy in curative intent.

OS was comparable between patients who underwent PM and SM: 82.3 versus 80.9 months; HR = 1.02, 95% CI: 0.69–1.49; *p* = 0.94 (Figure 4). Notably, patients who were not candidates for surgery or local treatment at any point during their course of treatment had inferior OS with a median of 25 months. To account for potential lead time bias, patients treated with PM and SM were compared using PS. Of note, PM and SM were associated with comparable PS: 78.7 versus 63.8 months; HR = 0.89; 95% CI: 0.60–1.31, *p* = 0.56 (Figure S1).

**FIGURE 5:**
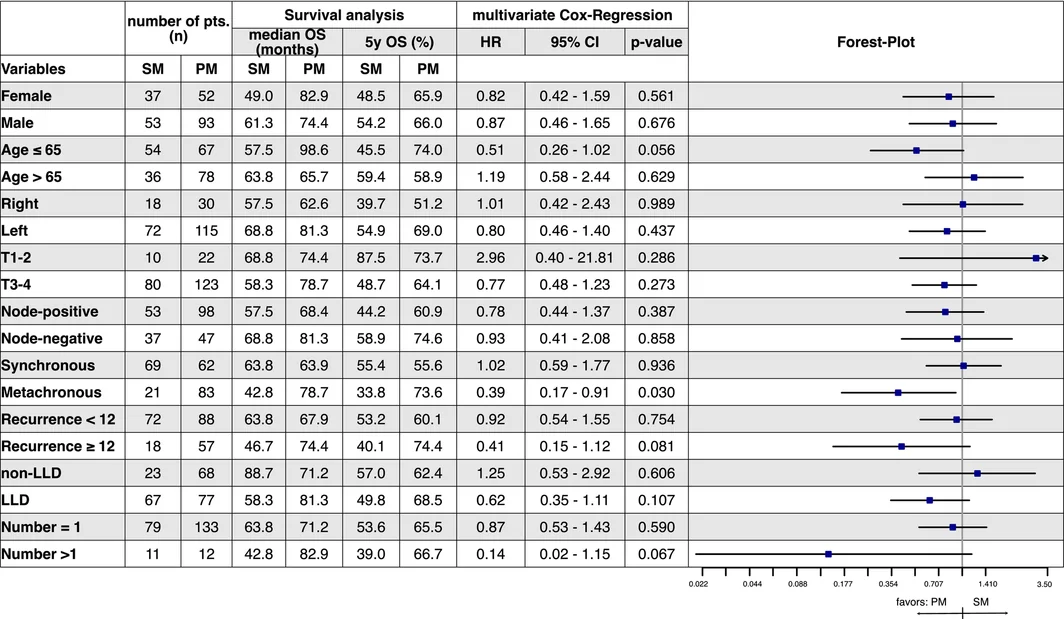
Forest plot of subgroup analyses comparing overall survival between treatment modalities (SM vs. PM). HRs with 95% CIs were derived from multivariable Cox proportional hazards regression models performed separately within each predefined subgroup. All Cox regression models were adjusted for the full set of covariates within the respective subgroup. Median overall survival (OS, months) and 5‐year OS rates (%) for each treatment group are provided for descriptive purposes. An HR < 1 indicates a survival benefit in favor of PM. 5y, 5 years; CI, confidence interval; HR, hazard ratio; LLD, liver‐limited disease; OS, overall survival; PM, primary metastectomy in curative intent; pts., patients; SM, secondary metastectomy in curative intent.

In multivariate Cox regression analysis, PM and SM led to comparable PS except for patients with metachronous metastasis favoring PM (HR 0.39; 95% CI: 0.17–0.91, *p* = 0.030) (Figure 5). A trend for increased PS after PM was observed in patients younger than 65 years (HR = 0.51; 95% CI: 0.26–1.02, *p* = 0.056), patients with tumor recurrence ≥ 12 months (HR = 0.41, 95% CI: 0.15–1.21, *p* = 0.081), and patients with LLD (HR = 0.62, 95% CI: 0.35–1.11, *p* = 0.107).

### 3.8 Subgroup Benefits of SM Compared NoLT Chemotherapy Only

In multivariate Cox regression analysis, all evaluated patient subgroups showed improved OS when eligible for SM compared NoLT patients who were never eligible for local treatment (Figure S2). However, this effect appeared to be mitigated in patients younger than 65 years (HR = 0.78, 95% CI: 0.40–1.54, *p* = 0.483) and patients with more than one metastatic site (HR = 0.69, 95% CI: 0.26–1.78, *p* = 0.437).

### 3.9 Survival of Patients Undergoing Metastasectomy Without Clearly Documented Curative Intent

Patients receiving metastectomy without clearly documented curative intention were excluded from the present analysis according to the inclusion criteria. Still, OS was evaluated in this subgroup as depicted in Figure S3.

### 3.10 Survival Analysis of Patients With Local Ablative Treatment

Patients who were treated with local ablative treatments were excluded due to challenges in evaluating treatment completeness retrospectively from the current analysis. Still, OS according to primary or secondary intervention is illustrated in Figure S4.

### 3.11 Treatment Effects Over Time

Given that the evaluated patient population spans a period of 23 years, we assessed the temporal influence on treatment efficacy by comparing PM versus PCT, with no indication of differences between the respective time periods (Table S5).

## 4 Discussion

This analysis investigates the relevance of metastasectomy with curative intent for long‐term outcome of patients with mCRC. While most previous studies focus on either initially resectable or irresectable mCRC [3, 10, 11, 12, 13], real‐world data enable a more comprehensive evaluation of PM and SM after chemotherapy response, helping to define treatment benefits in specific subgroups. Accordingly, we analyzed a well‐documented cohort treated at the CCC Munich^LMU^ between 2007 and 2021 [5].

Of 840 treated mCRC patients, 166 (20%) underwent PM and 109 (13%) received SM. These rates are consistent with resection frequencies from single‐site series (11%–54%) and exceed those in broader population‐based studies (1%–30%) [2, 3, 17, 18, 19, 20]. Further, our real‐world data perfectly mirror the results of a nation‐wide prospective intervention study with repeated centralized MDT assessment of resectability of 1086 Finnish mCRC patients (RAXO trial), which reported 21% and 9% for PM and SM, respectively [3].

PM was associated with significantly prolonged OS compared to first‐line chemotherapy (82.3 vs. 41.6 months; HR = 0.47; *p* < 0.0001), leading to a 10‐year OS rate of 32.2%. In contrast, patients receiving systemic therapy alone had a median OS of only 25 months. These survival outcomes align well with previous studies reporting survival times of 77.3–80.4 months and 10‐year OS rate of 16%–27% for resected patients versus a median OS of 20.8 months under chemotherapy alone [3, 12, 21, 22].

To minimize lead‐time bias, we compared PS for PM and SM. A comparable PS was observed between PM and SM (78.7 vs. 63.8 months, HR = 0.89; 95% CI: 0.60–1.31, *p* = 0.56). In literature, early findings suggest inferior outcome for SM^21^ as opposed to more recent data, which have illustrated similar survival for both PM and SM [3, 9, 12, 23, 24, 25, 26, 27, 28]. In line with recent publications, SM extended OS in our cohort as compared to patients treated with chemotherapy only (80.9 vs. 25.0 months; HR = 0.27, *p* < 0.0001). Here, the survival of patients receiving SM was comparable to the RAXO trial again (80.4 months) [3]. These results highlight the importance of reassessing resectability not only at diagnosis but also during systemic therapy to optimize outcomes.

Unlike previous studies limited to either resectable or unresectable mCRC, our analysis allows direct comparison of PM and SM within subgroups. Multivariate analysis confirmed largely equivalent survival benefits. However, patients with favorable features, such as metachronous metastasis, age ≤ 65, longer disease‐free interval, or liver‐limited disease, tended to benefit more from PM, consistent with guideline recommendations to prioritize surgery in prognostically favorable cases (e.g., based on Fong Score [29]) as opposed to starting with chemotherapy, when a favorable tumor biology is less probable [8, 30, 31].

The role of metastasectomy in high‐risk patients, such as those with right‐sided tumors, remains less clear. These patients more often present with aggressive histology and unfavorable metastatic patterns making them less likely to undergo curative resection [32, 33, 34, 35]. Here, we sought to determine whether a survival benefit from metastasectomy persists in patients with a priori inferior prognosis. In adjusted Cox regression analysis, a survival benefit from PM and SM, even in subgroups with poor prognostic features, was demonstrated. These findings support previous evidence, that appropriately selected high‐risk patients may still benefit from surgery [3, 36, 37, 38, 39].

We acknowledge limitations due to the retrospective, non‐randomized design, including potential for selection bias and incomplete molecular data (e.g., *RAS*/*BRAF* mutation status), which limited the feasibility of further subgroup analyses. Nevertheless, we examined available clinical surrogate parameters being associated with molecular alterations (e.g., primary tumor sidedness) and indicators of high‐risk disease (e.g., synchronous metastases, short recurrence interval).

Further, comparisons between PM, SM, and PCT are subject to inherent selection bias. Patients undergoing PM typically present with limited tumor burden and favorable metastatic patterns, whereas patients receiving SM constitute a biologically selected subgroup of initially unresectable mCRC, as eligibility for secondary resection requires sufficient response to systemic therapy. Despite multivariable adjustment, residual confounding due to unmeasured factors, including tumor biology and metastatic extent, cannot be fully excluded and should be considered when interpreting survival differences between treatment strategies.

To ensure consistency and data reliability, the analysis included only patients whose metastases had been surgically treated. Specifically, local ablation techniques (e.g., brachytherapy, microwave or radiofrequency ablation) were excluded due to challenges in evaluating treatment completeness retrospectively. While these approaches may be considered equivalent to surgical resection under certain conditions [31], their impact will be explored in future analyses. Still, we analyzed survival outcomes in patients undergoing primary and secondary interventional treatments. Notably, the inferior survival observed in patients treated with local ablation compared with those who underwent surgery likely reflects the fact that ablative approaches were predominantly applied in frail and inoperable patients with a poorer prognosis. Further evaluation of this patient subgroup is currently ongoing and will be reported separately.

To further enhance data quality, we implemented independent quality checks, used linkage with the Bavarian Cancer Registry to reduce censoring, and applied multivariate regression to adjust for confounders.

Despite its limitations, our use of real‐world data provides an important complement to clinical trial results by enabling the evaluation of curative‐intent metastasectomy in an unselected, routine‐care population. In particular, our findings expand upon the outcomes of the prospective RAXO trial by confirming similar resection rates and survival outcomes in a real‐world setting.

## 5 Conclusion

Our study underscores the importance of initial and repeated MDT assessments for identifying candidates for curative‐intent metastasectomy in mCRC. This strategy proved feasible in a real‐world setting at a high‐volume academic center and resulted in excellent long‐term outcomes across all evaluated prognostic subgroups. The alignment of our findings with prospective trials such as RAXO supports the broader applicability of MDT‐guided resection strategies throughout the treatment course of mCRC.

## Author Contributions

**Julian W. Holch:** conceptualization, methodology, data curation, investigation, validation, formal analysis, supervision, project administration, writing – original draft, writing – review and editing, resources. **Jo‐Ann Eisenburger:** methodology, data curation, investigation, validation, formal analysis, visualization, writing – review and editing, project administration. **Antony Weber:** data curation, formal analysis, investigation, validation, writing – review and editing. **Iris Kammermeier‐Wehlau:** data curation, validation, formal analysis, investigation, writing – review and editing. **Yannick Wiberg:** data curation, investigation, validation, formal analysis, writing – review and editing. **Fabian Allmendinger:** formal analysis, investigation, writing – review and editing. **Kathrin Heinrich:** investigation, formal analysis, writing – review and editing. **Lena Weiss:** formal analysis, investigation, writing – review and editing. **C. Benedikt Westphalen:** investigation, formal analysis, writing – review and editing. **Victoria Krenmayr:** investigation, formal analysis, writing – review and editing. **Klara Dorman:** formal analysis, investigation, writing – review and editing. **Danmei Zhang:** investigation, formal analysis, writing – review and editing. **Theres Fey:** investigation, formal analysis, writing – review and editing. **Karin Berger‐Thürmel:** investigation, formal analysis, writing – review and editing. **Ulrich Wirth:** investigation, formal analysis, writing – review and editing. **Florian Kühn:** investigation, formal analysis, writing – review and editing. **Michael von Bergwelt‐Baildon:** investigation, formal analysis, writing – review and editing. **Jens Werner:** investigation, formal analysis, writing – review and editing. **Volker Heinemann:** conceptualization, investigation, formal analysis, methodology, validation, supervision, writing – review and editing.

## Ethics Statement

Ethical approval was obtained prior to data processing by the LMU Ethical Review Board on 10.09.2021 under project number 21‐0741. In accordance with this approval, no explicit patient consent was obtained.

## Conflicts of Interest

Kathrin Heinrich has received honoraria from Amgen, BMS, Merck, MSD, Roche, Taiho, Servier, Takeda, and streamedup, has done consulting/served on advisory boards for Amgen, Servier, and Merck, and has received travel support/expenses from MSD, Amgen, Merck, Servier, and Takeda. Lena Weiss has received honoraria from Merck, Roche, and Servier, travel expenses from Merck and Amgen, and served on advisory boards for Merck and Roche. C. Benedikt Westphalen has received honoraria from Amgen, Bayer, BMS, Chugai, Celgene, Falk, GSK, Janssen, Ipsen, MSD, Merck, Roche, Servier, SIRTeX, and Taiho, served on advisory boards for Bayer, BMS, Celgene, Incyte, Janssen, Lily, MSD, Servier, Shire/Baxalta, Rafael Pharmaceuticals, RedHill, and Roche, has received travel support by Bayer, Celgene, Janssen, MSD, RedHill, Roche, Servier, and Taiho and research grants (institutional) by Roche and Deutsche Krebshilfe (institutional), is a member of the EU Commission expert group: Mission Board for cancer, is a member of the BMBF expert group: Forum Zukunftsstrategie, is a member of the BMBF steering committee: Strategiekreis Dekade gegen Krebs. All unrelated to the current study. Victoria Krenmayr has received travel expenses from Nordic Pharma and training costs from BMS. Klara Dorman reports honoraria from AstraZeneca and support for travel, accommodation, and expenses from Servier, GSK, Bristol Myers Squibb, and AstraZeneca. Danmei Zhang reports receiving honoraria from AstraZeneca and travel funding from AstraZeneca, Servier, and Amgen. Michael von Bergwelt‐Baildon was supported by AMGEN, Astellas, AstraZeneca, Bristol‐Myers Squibb, Daiichi Sankyo, KITE/Gilead Mologen, Miltenyi, MSD Sharp & Dohme, Novartis, Priothera, Roche, TABBY, and Vision Zero. Jens Werner is on the executive board of the German Society of Surgery (Deutsche Gesellschaft für Chirurgie, DGCH). Volker Heinemann reports receiving fees for talks and advisory board roles from Merck. Amgen, Roche, Sanofi, Servier, Pfizer, Pierre‐Fabre, AstraZeneca, BMS, MSD, Novartis Terumo, Oncosil, NORDIC, Seagen, GSK, and for receiving research funding from Merck, Amgen, Roche, Sanofi, Boehringer‐Ingelheim, SIRTEX, and Servier. Julian W. Holch served on an advisory board for Merck, Roche, Servier, Takeda, and Beigene, has received travel support from Novartis, Merck, Takeda, and Beigene, and has research funding from Merck. The other authors declare no conflicts of interest.

## Supporting information


**Table S1:** Number of metastases in the study population.
**Table S2:** First‐line treatment in patients receiving upfront chemotherapy.
**Table S3:** Type of primary metastectomy in curative intent.
**Table S4:** Type of secondary metastectomy in curative intent.
**Table S5:** Overall survival by initial treatment across treatment eras.
**Figure S1:** Post‐resection survival after primary versus secondary metastectomy.
**Figure S2:** Forest plot of subgroup analyses comparing overall survival between treatment.
**Figure S3:** Overall survival by elibility for metastectomy in or without curative intent.
**Figure S4:** Overall survival by elibibility for metastectomy or local ablative treatments.

## Data Availability

All source code is publicly available on GitHub: https://github.com/JoEisenburger/multivariate‐subgroup‐analysis/tree/main. Interested researchers should direct their scientific proposal to the corresponding author for approval and access to deidentified data. The shared data may only be used to achieve the aims of the approved proposal. Further information is available from the corresponding author upon reasonable request.
