# Supplementary material for: The Relevance of Curative‐Intent Metastasectomy in Colorectal Cancer Patients: Real‐World Insights From a Certified Comprehensive Cancer Center in Germany
Source: Int J Cancer. 2026 May 9;159(8):2025–35. doi: 10.1002/ijc.70540 (PMC13495870; doi:10.1002/ijc.70540)
Supplement: Supplementary file 1 [file IJC-159-2025-s001.pdf]

The Relevance of Curative-Intent Metastasectomy in Colorectal Cancer Patients: Real-World Insights from a Certified Comprehensive Cancer Center in Germany

Julian W. Holch, Jo-Ann Eisenburger, Antony Weber, Iris Kammermeier-Wehlau, Yannick Wiberg, Fabian Allmendinger, Kathrin Heinrich, Lena Weiss, C. Benedikt Westphalen, Victoria Krenmayr, Klara Dorman, Danmei Zang, Theres Fey, Karin Berger-Thürmel, Ulrich Wirth, Florian Kühn, Michael von Bergwelt-Baildon, Jens Werner, Volker Heinemann

Contents

SUPPLEMENTARY TABLES 2

TABLE S1: NUMBER OF METASTASES IN THE STUDY POPULATION 2

TABLE S2: FIRST-LINE TREATMENT IN PATIENTS RECEIVING UPFRONT CHEMOTHERAPY 3

TABLE S3: TYPE OF PRIMARY METASTECTOMY IN CURATIVE INTENT 4

TABLE S4: TYPE OF SECONDARY METASTECTOMY IN CURATIVE INTENT 5

TABLE S5: OVERALL SURVIVAL BY INITIAL TREATMENT ACROSS TREATMENT ERAS 6

SUPPLEMENTARY FIGURES 7

FIGURE S1: POST-RESECTION SURVIVAL AFTER PRIMARY VERSUS SECONDARY METASTECTOMY 7

FIGURE S2: FOREST PLOT OF SUBGROUP ANALYSES COMPARING OVERALL SURVIVAL BETWEEN TREATMENT MODALITIES (NOLT VS. SM) 8

FIGURE S3: OVERALL SURVIVAL BY ELIBILITY FOR METASTECTOMY IN OR WITHOUT CURATIVE INTENT 9

FIGURE S4: OVERALL SURVIVAL BY ELIBIBILITY FOR METASTECTOMY OR LOCAL ABLATIVE TREATMENTS 10

Supplementary Tables

Table S1: Number of metastases in the study population

	Total Cohort (n = 840)		PM (n = 166)		PCT (n = 520)	
	N	%	N	%	N	%
**Liver**						
Liver total	604	71.9	93	56.0	407	78.3
Liver only	397	47.3	85	51.2	245	47.1
Liver + Lung	72	8.6			65	12.5
**Lung**						
Lung total	200	23.8	20	12.0	148	28.5
Lung only	48	5.7	19	11.4	23	4.4
**Peritoneum**						
Peritoneum total	130	15.5	9	5.4	82	15.8
Peritoneum only	44	5.2	2	1.2	30	5.8
**Lymph node (distant)**						
Lymph node total	108	12.9	11	6.6	85	16.3
Lymph node only	24	2.9	5	3.0	17	3.3
**Locoregional recurrence**	52	6.2	35	21.1	9	1.7
**Ovary**	27	3.2	11	6.6	8	1.5
**Bone**	26	3.1			19	3.7
**Brain**	13	1.5	3	1.8	1	0.2
**Others**	23	2.7			13	2.5

Abbreviations: PCT: primary chemotherapy, PM: primary metastectomy in curative intent.

Table S2: First-line treatment in patients receiving upfront chemotherapy

**Chemotherapy**	N	%
FOLFOX	189	36.3
FOLFIRI	181	34.8
FOLFOXIRI	42	8.1
Capecitabin	35	6.7
CapOx	29	5.6
5-Fluorouracil + Folinic acid	15	2.9
CapIri	13	2.5
Others	16	3.1
**Monoclonal Antibodies**		
None	132	25.4
anti-VEGF	247	47.5
anti-EGFR	140	26.9
others	1	0.2

Abbreviations: anti-EGFR: anti-epidermal growth factor receptor; anti-VEGF: anti-vascular endothelial growth factor; CapIri: Capecitabin, irinotecan; CapOx: Capecitabin, oxaliplatin; FOLFIRI: 5-Fluorouracil, folinic acid, irinotecan; FOLFOX: 5-Fluorouracil, folinic acid, oxaliplatin; FOLFOXIRI: 5-Fluorouracil, folinic acid, oxaliplatin, irinotecan.

Table S3: Type of Primary Metastectomy in Curative Intent

Metastectomy	N (%)
**Liver resection**	**93 (56.0 %)**
Hemihepatectomy	14 (8.4 %)
Extended hemihepatectomy	4 (2.4 %)
Partial liver resection	71 (42.8%)
Partial liver resection + lymphadenectomy	3 (1.8 %)
Partial liver resection + adnexectomy	1 (0.6%)
**Peritonectomy**	**32 (19.3 %)**
Peritonectomy	4 (2.4 %)
Peritonectomy + adnexectomy	2 (1.2 %)
Resection of locoregional recurrence	26 (15.7 %)
**Lung resection**	**20 (12.0 %)**
Partial lung resection	17 (10.2 %)
Partial lung resection + lymphadenectomy	3 (1.8%)
**Distant lymphadenectomy**	**5 (3.0 %)**
**Adnexectomy**	**6 (3.6 %)**
**Brain resection**	**3 (1.8 %)**
**Others**	**7 (4.2 %)**
**Total**	**166 (100 %)**

Table S4: Type of Secondary Metastectomy in Curative Intent

Metastectomy	N (%)
**Liver resection**	**89 (81.7 %)**
Hemihepatectomy	26 (23.9 %)
Extended hemihepatectomy	22 (20.2 %)
Partial liver resection	41 (37.6 %)
**Lung resection**	**7 (6.4 %)**
Partial lung resection	6 (5.5 %)
Partial lung resection + lymphadenectomy	1 (0.9 %)
**Peritonectomy**	**4 (3.7 %)**
Peritonectomy	3 (2.8 %)
Peritonectomy + adnexectomy	1 (0.9 %)
**Distant lymphadenectomy**	**2 (1.8%)**
**Combined resections**	**7 (6.4 %)**
Partial hepatectomy + partial lung resection	3 (2.8 %)
Hemihepatectomy + peritonectomy	2 (1.8 %)
Partial liver resection + peritonectomy	2 (1.8 %)
**Total**	**109 (100 %)**

Table S5: Overall survival by initial treatment across treatment eras

		Number of pts.		Median Os (months)				
**Groups**	**Timelines**	**PCT**	**PM**	**PCT**	**PM**	**HR**	**95% CI**	**P**
**Interval I**	Feb 1998 – Aug 2010	134	38	57.8	100.1	2.06	1.22 – 3.50	0.007
**Interval II**	Aug 2010 – Jul 2014	134	37	34.8	83.2	1.93	1.14 – 3.30	0.015
**Interval III**	Jul 2014 – Jan 2018	123	48	40.6	70.8	2.12	1.27 – 3.54	0.004
**Interval IV**	Jan 2018 – Jun 2021	129	43	30.5		2.34	1.19 – 4.58	0.013

Abbreviations: CI: confidence interval, HR: hazard ratio, OS: overall survival, PCT: primary chemotherapy, PM: primary metastectomy in curative intent, pts: patients.

Supplementary Figures

Figure S1: Post-resection survival after primary versus secondary metastectomy

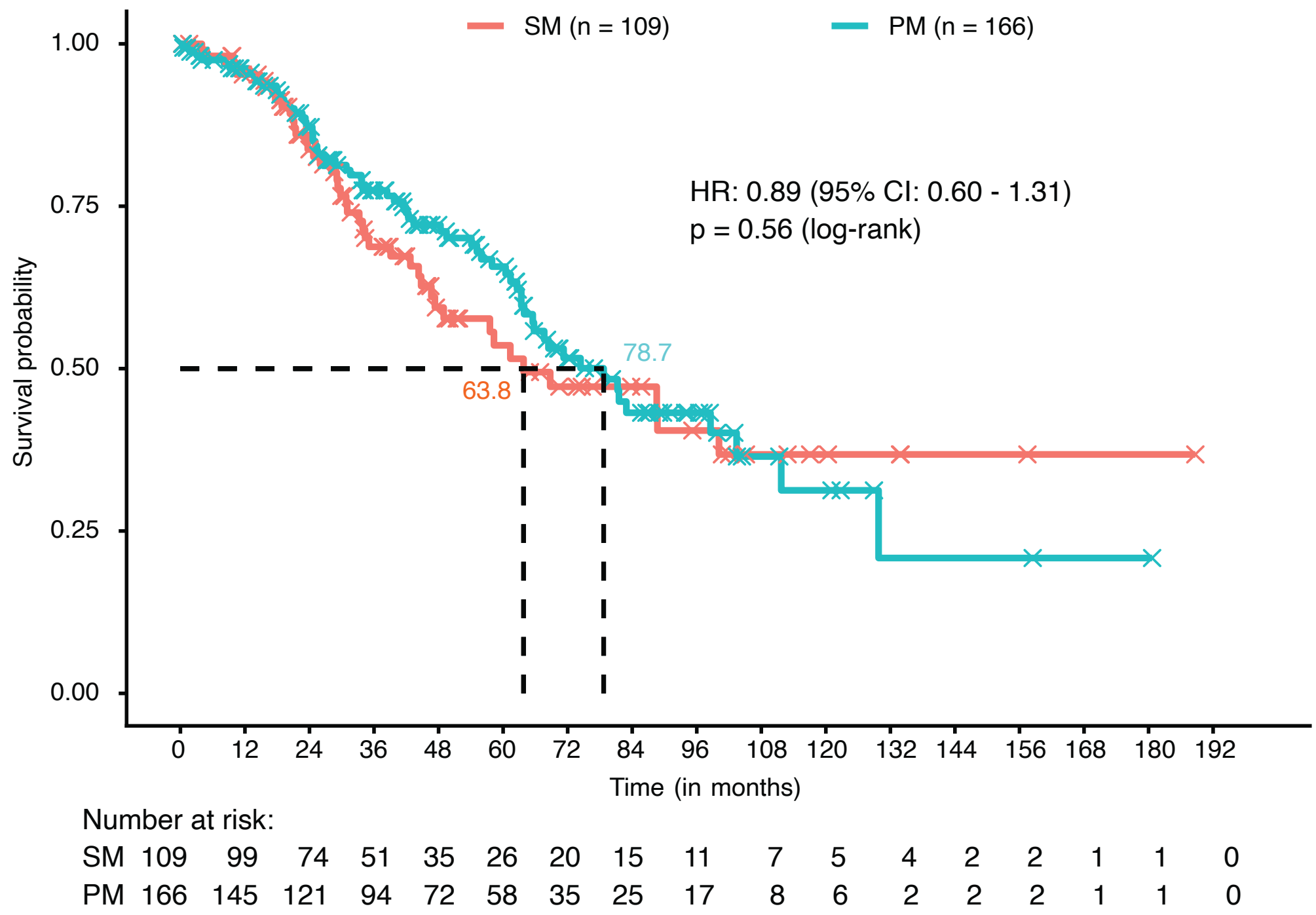

Abbreviations: CI: confidence interval, HR: hazard ratio, PM: primary metastectomy in curative intent, SM: secondary metastectomy in curative intent.

Figure S2: Forest plot of subgroup analyses comparing overall survival between treatment modalities (NoLT vs. SM)

	number of pts. (n)		Survival analysis				multivariate Cox-Regression		
			median OS (months)		5y OS (%)		HR	95% CI	p-value
Variables	NoLT	SM	NoLT	SM	NoLT	SM			
Female	60	37	26.7	53.5	16.8	49.5	0.36	0.17 - 0.73	0.005
Male	93	53	33.9	72.0	27.4	64.7	0.35	0.19 - 0.64	0.001
Age ≤ 65	73	54	42.5	62.8	32.8	53.7	0.78	0.40 - 1.54	0.483
Age > 65	80	36	30.0	80.9	16.7	65.7	0.16	0.07 - 0.36	< 0.001
Right	54	18	18.0	62.8	19.1	58.0	0.25	0.10 - 0.59	0.002
Left	99	72	36.8	80.9	27.0	59.1	0.36	0.21 - 0.61	< 0.001
T1-2	10	10	61.7	80.9	53.3	87.5	0.00	0.00 - 0.00	< 0.001
T3-4	143	80	31.7	68.3	21.6	56.1	0.37	0.24 - 0.59	< 0.001
Node-positive	127	53	32.1	65.5	23.5	55.6	0.41	0.24 - 0.71	0.001
Node-negative	26	37	30.5	80.9	28.0	61.2	0.22	0.09 - 0.52	0.001
Synchronous	100	69	30.5	80.9	27.5	63.4	0.32	0.19 - 0.54	< 0.001
Metachronous	53	21	34.8	50.2	17.5	35.5	0.41	0.17 - 0.98	0.045
Recurrence < 12	125	72	30.5	72.0	27.2	61.6	0.39	0.24 - 0.64	< 0.001
Recurrence ≥ 12	28	18	34.8	58.5	10.6	40.9	0.16	0.04 - 0.56	0.004
non-LLD	102	23	32.1	101.4	26.2	58.3	0.41	0.19 - 0.87	0.020
LLD	51	67	31.7	68.3	21.3	58.4	0.29	0.16 - 0.52	< 0.001
Number = 1	89	79	33.0	72.0	25.0	61.7	0.31	0.18 - 0.51	< 0.001
Number >1	64	11	30.5	58.5	24.1	39.0	0.69	0.26 - 1.78	0.437

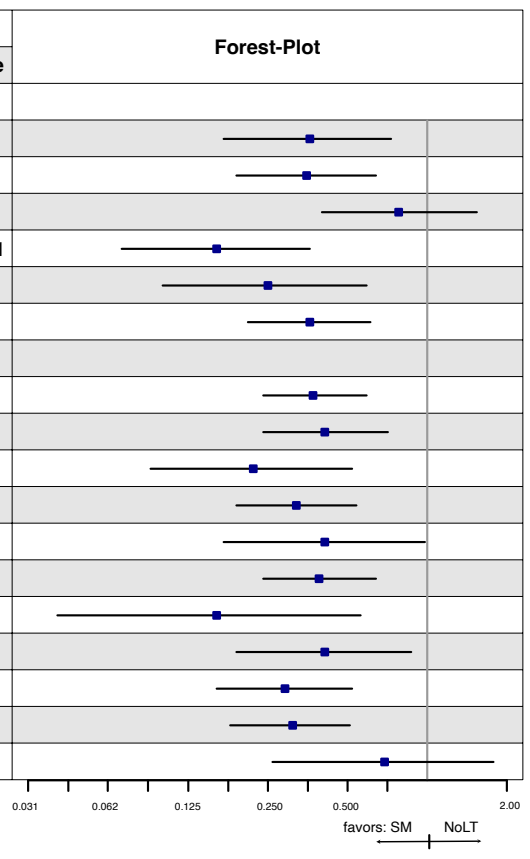

Hazard ratios (HRs) with 95% confidence intervals (CIs) were derived from multivariable Cox proportional hazards regression models performed separately within each predefined subgroup. All Cox regression models were adjusted for the full set of covariates within the respective subgroup. Median overall survival (OS, months) and 5-year OS rates (%) for each treatment group are provided for descriptive purposes. An HR < 1 indicates a survival benefit in favour of SM.

Abbreviations: 5y: 5 years, CI: confidence interval, HR: hazard ratio, LLD: liver-limited disease, NoLT: no local treatment, OS: overall survival, pts: patients, SM: secondary metastectomy in curative intent.

Figure S3: Overall survival by elibility for metastectomy in or without curative intent

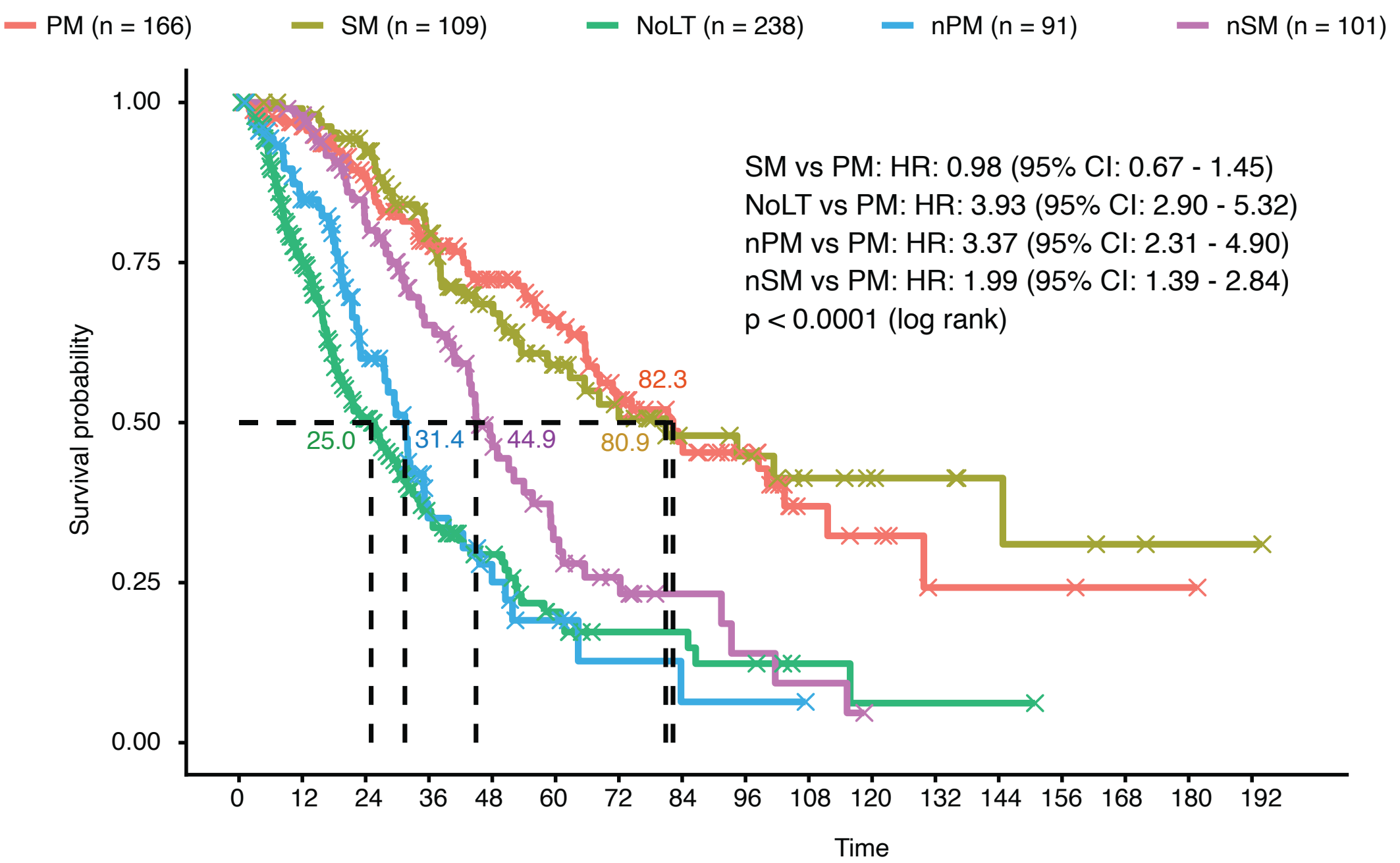

Number at risk:

	0	12	24	36	48	60	72	84	96	108	120	132	144	156	168	180	192	204
PM	166	148	124	98	75	59	39	28	20	8	6	2	2	2	1	1	0	
SM	109	106	93	70	47	31	24	16	14	9	7	6	4	3	2	1	1	0
NoLT	238	150	84	41	25	13	7	7	5	2	1	1	1	0				
nPM	91	69	37	15	9	5	2	1	1	0								
nSM	101	98	68	45	28	17	10	5	3	2	0							

Abbreviations: CI: confidence interval, HR: hazard ratio, NoLT: no local treatment, nPM: primary metastectomy without (documented) curative intent, nSM: secondary metastectomy without (documented) curative intent, PM: primary metastectomy in curative intent, SM: secondary metastectomy in curative intent.

Figure S4: Overall survival by elibibility for metastectomy or local ablative treatments

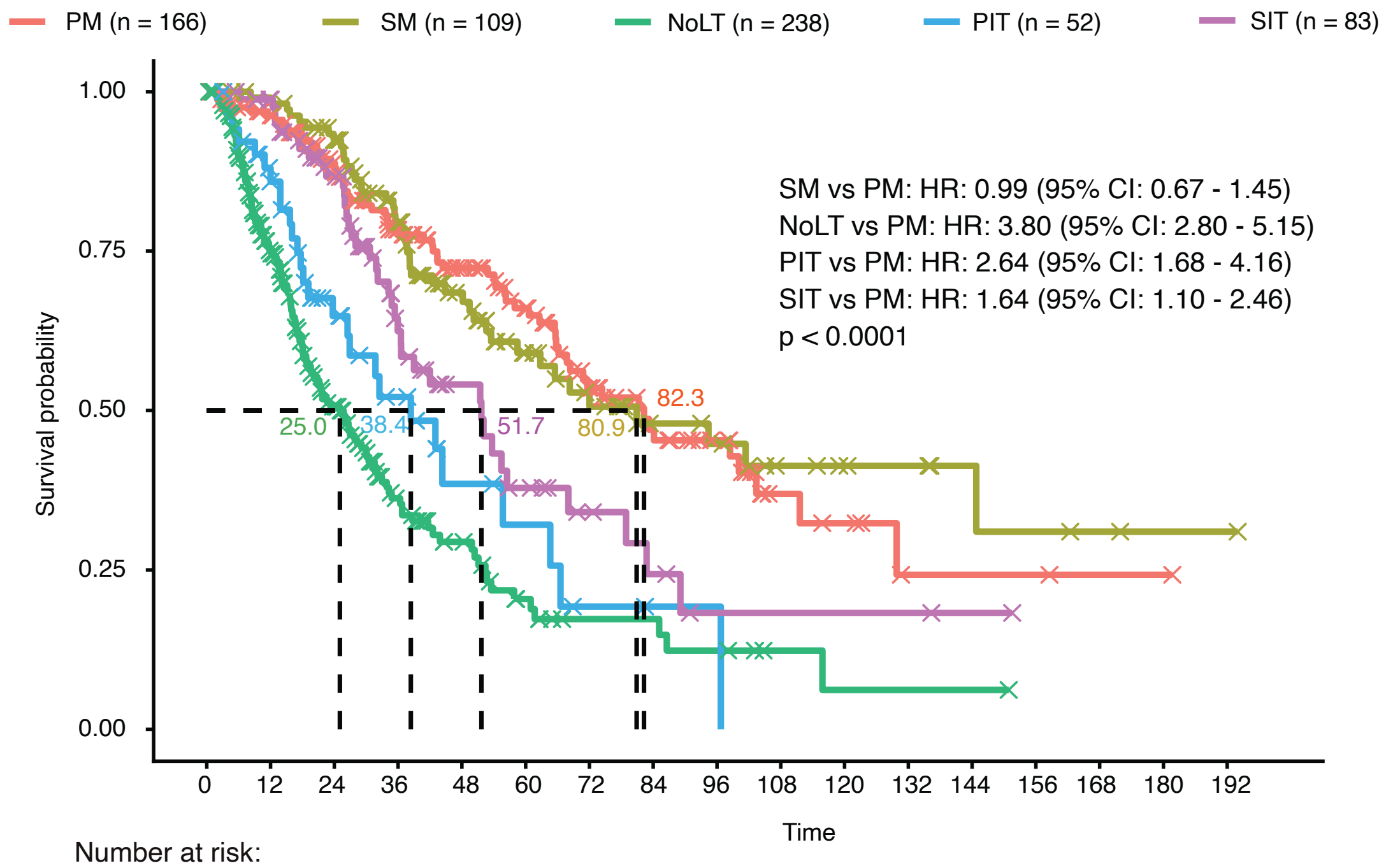

Number at risk:

PM	166	148	124	98	75	59	39	28	20	8	6	2	2	2	1	1	0	
SM	109	106	93	70	47	31	24	16	14	9	7	6	4	3	2	1	1	0
NoLT	238	150	84	41	25	13	7	7	5	2	1	1	1	0				
PIT	52	41	23	15	7	5	2	1	1	0								
SIT	83	78	58	31	20	13	8	5	2	2	2	2	1	0				

Abbreviations: CI: confidence interval, HR: hazard ratio, NoLT: no local treatment, PIT: primary interventional treatment, PM: primary metastectomy in curative intent, SIT: secondary interventional treatment, SM: secondary metastectomy in curative intent.
